# Geographic Variation in Age Structure and Longevity in the Nine-Spined Stickleback (*Pungitius pungitius*)

**DOI:** 10.1371/journal.pone.0102660

**Published:** 2014-07-15

**Authors:** Jacquelin DeFaveri, Takahito Shikano, Juha Merilä

**Affiliations:** Ecological Genetics Research Unit, Department of Biosciences, University of Helsinki, Helsinki, Finland; University of Basel, Switzerland

## Abstract

Variation in age and size of mature nine-spined sticklebacks (*Pungitius pungitius*) within and among 16 Fennoscandian populations were assessed using skeletochronology. The average age of individuals in a given population varied from 1.7 to 4.7 years. Fish from pond populations were on average older than those from lake and marine populations, and females tended to be older than males. Reproduction in marine and lake populations commenced typically at an age of two years, whereas that in ponds at an age of three years. The maximum life span of the fish varied from 3 to 7 years. Mean body size within and among populations increased with increasing age, but the habitat and population differences in body size persisted even after accounting for variation in population age (and sex) structure. Hence, the population differences in mean body size are not explainable by age differences alone. As such, much of the pronounced intraspecific variation in population age structure can be attributed to delayed maturation and extended longevity of the pond fish. The results are contrasted and discussed in the context of similar data from the three-spined stickleback (*Gasterosteus aculeatus*) occupying the same geographic area.

## Introduction

Early maturation at small size is usually accompanied by short life span, whereas late maturation at larger size is typically associated with long life span [1], [2]. This general life history pattern is thought to be driven mainly by variation in extrinsic mortality rates [2]. In general, individuals in populations subject to high extrinsic mortality rates as adults are expected to mature and reproduce at an early age and thus exhibit also small size and short longevity [3]–[6]. In contrast, populations subject to low extrinsic mortality rates may be selected for delaying their maturation at larger size, and consequently exhibit also prolonged lifespans [2]–[6].

While these general life history patterns have been demonstrated in numerous studies, there is also a great deal of variation around them (e.g. [7]–[8]). One possible source of this variation is that what triggers or constrains maturation varies among organisms or populations (e.g. [9]). If maturation is solely triggered by reaching a certain age, unfavorable environmental conditions will result in a population of small sized adults. If maturation is instead triggered by attaining a certain size, then unfavorable environmental conditions will delay maturation [9]. In fact, in some species both size and age appear to influence maturation probability [10], whereas in others neither of them are good predictors of maturation [11]–[13], (see also [14] and references therein). Similarly, the patterns of longevity associated with size and age at maturation are highly variable among organisms [15].

Many studies have investigated variation in age and size structuring of teleost fish populations (e.g. [3], [4], [16], [17]). However, most of these have been focused on economic/commercial interest, in relation to how the factors influencing age/longevity and size at maturation affect fisheries yields. In contrast, less attention has been focused on fish species of lower economic/commercial interest (but see: e.g. [18], [19]), such as those belonging to the family Gasterostidae, which are popular models in evolutionary ecology research [20]–[22]. The most thoroughly studied species of this family in this regard is the three-spined stickleback (*Gasterosteus aculeatus*; [23], [24]), but much less is known about the nine-spined stickleback (*Pungitius pungitius*). From the few studies that have investigated age and maturation in nine-spined sticklebacks, surveys of European populations have revealed that this species can reach ages of up to five year old [25]–[29], whereas Herczeg et al. [30] found individuals as old as seven years in northern Europe. Griswold & Smith [31] found individuals as old as five years old from Lake Superior, Gallagher & Dick [32] reported several three and four year olds from Baffin Island, yet Coad & Power [33] did not encounter any individuals older than two years after screening several hundreds of individuals from a Canadian river and lake. Moreover, while some populations begin spawning at an age of one year [25]–[28], others appear to delay maturation until an age of two years [30], [33]. However, due to the limited sampling of individuals and populations from different habitat types, the general picture about of life history trait variation among nine-spined stickleback populations is still fragmented.

The aim of this study was to explore variation in age structure, longevity and size-at-age within and among 16 different nine-spined stickleback populations, including marine and freshwater (cf. pond and lake) sites. To this end, skeltochronological methods were used to age a large number of individuals (n = 517; mean = 32 individuals/population) from Fennoscandia. Of particular interest was to explore whether there are consistent habitat differences in maturation age, average age of breeding individuals and their longevity, as well as whether size-at-age distributions, and hence growth trajectories, are similar in different habitats. Given that the studied pond populations lack predatory fish, and hence presumably also experience reduced extrinsic mortality rates, we expected to find higher average age and longevity in pond, as compared to lake and marine populations with predatory fish.

## Materials and Methods

### Ethics statement

The research described in this paper was conducted in strict accordance with the Finnish, Russian and Swedish legislation. The fish were collected under appropriate national fishing licenses of the respective countries. According to The Act of Animal Experimentation (FINLEX 497/2013; http://www.finlex.fi/fi/laki/alkup/2013/20130497), the conducted research does not classify as animal experiments. The fish were sacrificed by an overdose of MS-222 (tricaine methanesulfonate) immediately upon their capture. Hence, suffering before anesthesia was minimal.

### Study sites and sampling

**Figure 1:**
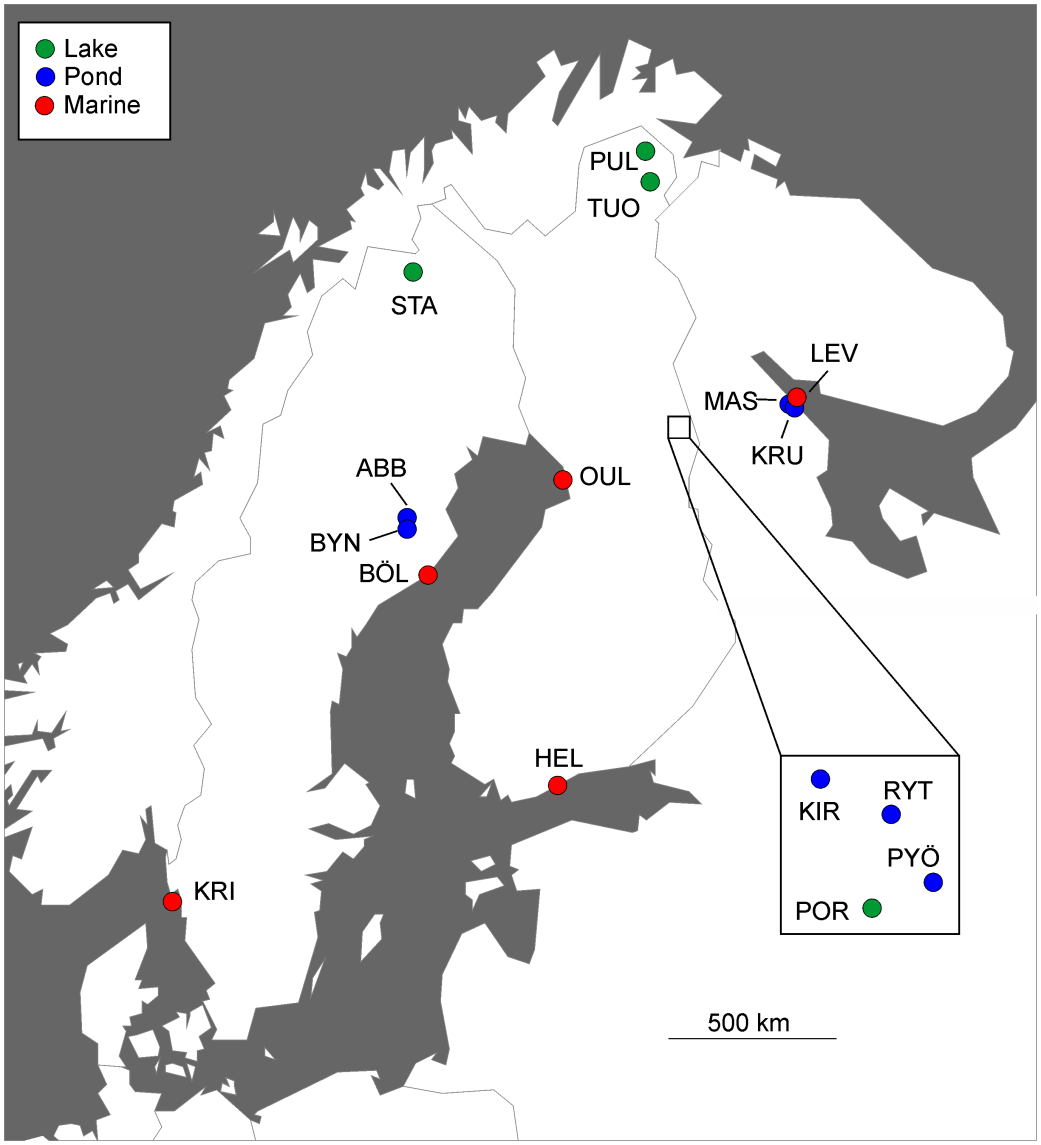
A map showing the 16 sampling locations in Fennoscandia. Green = lakes; blue = ponds; red = marine.

Adult fish were collected from five marine and eleven freshwater sites situated in different parts of Fennoscandia (Fig. 1; Table 1). The marine sites covered three different sea areas (*viz*. North Sea, Baltic Sea and White Sea), and the freshwater sites can be classified as belonging to either White Sea, Baltic Sea or Atlantic drainages (Table 1). Additional information about the physical and biological characteristics of the study sites are given in Table S1. From each locality an average of 32 individuals (range 15–80) were aged, and the total number of aged individuals was 517 (Table 1). In most cases, roughly a 1∶1 sex-ratio was available, except Lake Iso-Porontima (POR) from which only one female was obtained (Table 1). Data from seven populations (marked with ‘*’ in Table 1) are partly the same as used in Herczeg et al. [30], but supplemented here with additional individuals from these localities.

**Table 1:** Descriptive information about the study sites and sample sizes.

						Sample size (n)		
Location	Code	Coordinates	Habitat	Drainage	Collection date	Females	Males	Total†
Fiskebäckskil	KRI	58°24′N, 11°47′E	Marine	Atlantic	Jun 2009	18	12	30
Helsinki	HEL*	60**°**13′N, 25**°**11′E	Marine	Baltic Sea	Jun 2006	15	22	37
Oulu	OUL*	64°58′N, 25°29′E	Marine	Baltic Sea	Jun 2003	18	14	32
Bölesviken	BÖL	63°39′N, 20°12′E	Marine	Baltic Sea	May 2007	15	15	30
Levin Navolok	LEV	66**°**18′N, 33**°**25′E	Marine	White Sea	Jun 2006	12	12	24
Pulmankijärvi	PUL	69°58′N, 27°58′E	Lake	Atlantic	Jun 2003	21	33	62
Tuolpujärvi	TUO*	69**°**34′N, 28**°**02′E	Lake	Atlantic	Sept 2003	11	16	35
Iso-Porontima	POR	66**°**12′N, 29**°**16′E	Lake	White Sea	Jun 2006	14	1	15
Stavlussukiavri	STA	68°06′N, 20°01′E	Lake	Baltic Sea	July 2008	15	16	31
Pyöreälampi	PYÖ*	66**°**15′N, 29**°**26′E	Pond	White Sea	Jun 2006	17	18	35
Rytilampi	RYT*	66**°**23′N, 29**°**19′E	Pond	White Sea	Jun 2006	17	17	34
Kirkasvetinen lampi	KIR	66**°**26′N, 29**°**08′E	Pond	White Sea	Jun 2006	15	15	30
Krugloje	KRU*	66**°**18′N, 33**°**25′E	Pond	White Sea	Jun 2006	18	11	29
Mashinoje	MAS	66**°**18′N, 33**°**25′E	Pond	White Sea	Jun 2006	13	17	30
Abbortjärn	ABB	64**°**29′N, 19**°**26′E	Pond	Baltic Sea	May 2007	14	16	30
Bynastjärnen	BYN*	64**°**27′N, 19**°**26′E	Pond	Baltic Sea	May 2007	17	16	33
Total						250	251	517

Populations marked with asterix have been subject to an earlier [28] skeletochronological study.

† Includes 16 non-sexed individuals.

With the exception of Lake Tuolpujärvi (TUO), fish (cf. Table 1) were collected during early breeding season (April to July) between 2003 and 2009 either using minnow traps or seine nets. The collection was originally made mainly for purposes of population genetic investigations and to obtain brood stock for breeding experiments. Hence, collection was aimed to select only mature and/or reproductive individuals. Although no other restrictions were applied to the sampling – hence a random sample was obtained for most populations – several immature individuals were encountered in Lake Pulmanki (PUL) and were subsequently excluded from comparisons of age at maturation. Most fish were initially sexed (for other studies) phenotypically by external examination (i.e. females were identified by enlarged abdomens, males identified by nuptial coloration). These conditions also verified maturity for the current study. In the case of uncertainty, lateral incisions were made for gonadal inspection. In some cases when phenotypic sex or gonads were unavailable (n = 67), genotypic sex was determined using a sex-specific microsatellite locus (Stn19) as explained in Shikano et al. [34]. A subset of samples for which phenotypic and genotypic sex were available (n = 219) confirmed a high degree of concordance between methods (96.9% agreement). We note that most (61.1%) of the mismatches occurred in individuals belonging to the Western European lineage of nine-spined sticklebacks [35] for which the Stn19 locus does not give reliable information about sex due to chromosomal re-arrangement [36]. However, those individuals for which sex was only determined by genotype were from the Eastern European lineage, therefore the genotypic sex was reliable.

### Age and size determination

The aging was conducted using skeltochronological methods as detailed in Herczeg et al. [30] and DeFaveri & Merilä [37]. In short, fin ray cross-sectioning (e.g. [38]) was used, and this was verified to yield reliable results by comparison to otolith based age-determination performed for a sub-sample of individuals aged with both methods. Dorsal, pectoral or pelvic fins were first cut near the fin-base, cleaned from extra tissue, and treated with 1,2-Propanediol to gain better contrast. After air-drying, fins were stained with a neutral red solution (with acetic acid) and the annuli (‘lines or arrested growth’) were evaluated under a microscope with 30–100× magnification.

Age of the individuals was estimated as the number of visible annuli assuming that the fish with one annulus would have been born the year before, and would therefore be second calendar year (yearling) individuals. Similarly, a fish with two annuli would be in the third calendar year (a two year old fish), and so forth. Assuming random and representative sampling, a conservative estimate for maximum age of fish ( = longevity) at each site was obtained as the age of the oldest individual in the sample. This is a conservative estimate because of the relatively small sample sizes per locality. Likewise, the age at maturation was estimated from the age of the youngest reproductively active individual in the given sample. Since there was uncertainty of reproductive status of some individuals in the samples, and some immature (i.e. young of the year) individuals in Lake Pulmanki (PUL) samples, these individuals were omitted from the estimation of age at maturation. One person (Mika Vinni) well experienced in fish aging methods performed all aging.

Apart from age determination, standard length of each fish was measured to the nearest 0.1 mm under a stereomicroscope. The same person measured all the fish. Before taking the measurements, all fish had been stored in alcohol for several (3 to 10) years. Although storage in alcohol can cause shrinkage, this should be of minor concern for two reasons. First, most of this shrinkage takes place within the first two months following preservation (e.g. [39], [40]), and all measurements were taken at least after 36 months of preservation. Second, the shrinkage has been found to be minor (<3%) in other comparable sized fish [40].

### Statistical analyses

The age and size data was analyzed with generalized linear models. Individual age was fitted as normally distributed response variable, with habitat type (marine, lake or pond population) and sex as fixed factors, and the population identity (nested within habitat type) as a random factor. Habitat*sex interaction was also fitted. Qualitatively similar results were obtained if age was fitted as a Poisson distributed response variable, and/or if the population (Lake Iso-Porontima; POR) with only one male was excluded.

To investigate how mean body size is related to age, a model was fitted where individual size (a normally distributed response variable) was a function of habitat type (fixed factor), sex (fixed factor) and population (random factor nested within habitat type). To see whether the size differences persisted after accounting for variation due to age, this analysis was repeated by including age as a covariate into the model. In the case where size differences are due to age differences among habitats and populations, the expectation is that there should be no significant habitat or population effects after accounting for age effects. In all these models, sex*habitat and sex*population (within habitat type) interactions were also included, but dropped from the final models if non-significant.

In the case of both age and size analyses, we also conducted analyses where drainage (three levels; see Table 1) was fitted as fixed factor (Habitat type and Population becoming nested within it). Since it never explained any significant amount of variation in data (or influenced the inference about other effects in the model), we only report results for models excluding the drainage effect. However, since we sampled different localities only once, we assume that the possible temporal variation in population age and size structure is random in respect to habitat type and drainage. This seems a reasonable assumption in light of data from earlier studies of stickleback age and size structure [27], [41]. Moreover, would this assumption be violated, the likely effect is to make our tests of habitat effects conservative.

Statistical analyses were performed with JMP 10 Pro (ver. 10.0.2d1) statistical package (SAS Institute Inc.).

### Data accessibility

All the data behind this publication has been submitted to a Dryad archive: [DOI: to be inserted upon acceptance].

## Results

**Figure 2:**
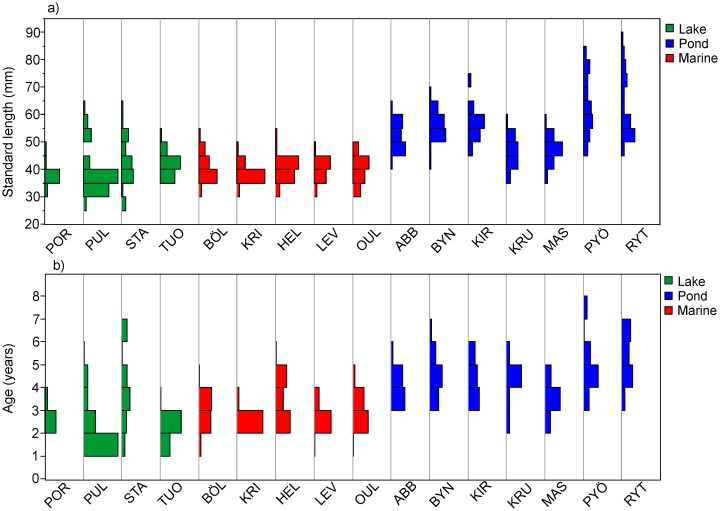
Distribution of (a) body size (standard length) and (b) age of nine-spined sticklebacks in different localities. Green = lakes; blue = ponds; red = marine.

The age of the youngest mature fish in a given population ranged from one to three years, and the oldest fish from three to seven years (Fig. 2). Six-year-old individuals were encountered from three pond and one lake population. The oldest individual from the marine habitat was five years old, though the oldest marine fish were more commonly four years old (Fig. 2).

The mean age of the fish differed significantly among habitat types (GLMM, Habitat: F_2,13.16_ = 14.56, P<0.005): fish from the marine and lake populations were younger than the fish from the pond populations (Tukey HSD, P<0.05), but there was no significant difference between lake and marine fish (Tukey HSD, P>0.05; Fig. 2). Females were on average older than males (GLMM, Sex: F_1,487.8_ = 34.08, P<0.001), but there was no evidence that this difference would be habitat dependent (GLMM, sex*habitat; F_2,487_ = 0.03, P = 0.96). However, even after accounting for habitat effects, the random effect of the population identity was significant, revealing among-population heterogeneity in mean age within habitat types (variance component: 0.315; S.E. = 0.133; 29.8% of variance explained).

The mean size of the fish differed significantly (GLMM, Habitat: F_2,13.06_ = 18.22, P = 0.002) among habitat types: pond fish were larger than lake or marine fish (Tukey’s HSD, P<0.05; Fig. 2). However, the difference between lake and marine populations was not significant (Tukey’s HSD, P>0.05). Females were on average larger than males (GLMM, Sex: F_1,479.3_ = 53.66, P<0.001), but the degree of sexual dimorphism differed among habitat types as shown by significant sex*habitat interaction (F_2,478.8_ = 3.63, P = 0.027). The latter effect came about because the degree of sexual size dimorphism was more pronounced in ponds than in other habitat types, as was also shown earlier [38]. There was also a lot of variation in size among populations within the habitat type, as revealed by the random effect of population identity, which explained 38.7% variation in the data (variance component estimate 22.51, S.E. = 9.29). Part of this within-habitat variation could be attributed to the fact that one lake population (Lake Tuolpujärvi; TUO) was sampled later in the year than the other populations (Table 1). However, the results and inferences remain qualitatively similar if this population is excluded from the analyses.

**Figure 3:**
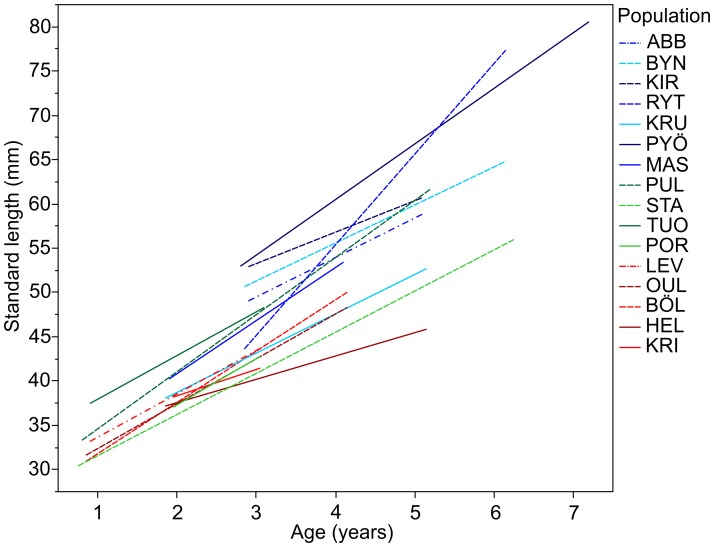
Mean body size of nine-spined sticklebacks as a function of age in different localities. Green = lakes; blue = ponds; red = marine.

All the results above remained qualitatively similar and statistically significant when age (F_1,484.4_ = 581.30, P<0.001) was added into the model as covariate, suggesting that the size differences among populations, sexes, and habitats were not caused solely by variation in age. This was true also when allowing for the relationship between age and size to differ among habitat types (habitat*age; F_2,482.4_ = 4.92, P = 0.008) or between sexes (sex*age; F_1,472.2_ = 4.52, P = 0.034). The latter two significant interactions revealed that size increases with age was faster for pond than for marine and lake populations (Fig. 3), and for females than for males. The visualization of the size as a function of age in different populations illustrates the main conclusion in respect to size: for a given age, the pond fish are on average much larger than the marine or lake fish (Fig. 3).

## Discussion

The results demonstrate marked habitat-specific differentiation in age at maturation, longevity and size-at-age in northern European nine-spined sticklebacks. The overall patterns were clear: individuals in most pond populations mature late, live longer and reach larger body size, whereas the opposite is true for most marine and lake populations. However, exceptions to this pattern were observed in all habitat types, highlighting the importance of adequate sampling in identifying deviations from generalizations. Nevertheless, one consistent trend was that large size-at-age was confined only to the pond habitat. In the following, we will discuss these observations and relate them to what is known about life history variation in nine- and three-spined sticklebacks, as well as address some implications of these findings on future studies.

Age of nine-spined sticklebacks has received little attention in past studies, and variation among populations has generally not been well characterized due to low sample sizes (e.g. one or two populations; [25], [26], [28], [31], [33]). A recent study by Herczeg et al. [30] included seven Fennoscandian populations, which, until the present study, represented the largest number of populations screened. However, when categorized by habitat type, the number of populations within habitat was still relatively low (e.g. one lake population, two marine populations). Here, we have increased both the number of populations within habitat as well as the number of individuals within population to provide the most comprehensive sampling to date. This allowed us to identify habitat-specific patterns of variation in longevity, size, and age-at-maturation. For example, our increased numerical and geographic coverage allowed us to confidently confirm that the maximum life span among Fennoscandian nine-spined sticklebacks is at least seven years. While several five to six year old individuals were found from nearly all freshwater populations, only one five year old individual was found in one marine population – the rest were four and under (mean = 2.4 years). Coupled with the findings of Griswold & Smith [31], who also found several four and five year olds in Lake Superior, it becomes clear that there is a wider degree of variation in longevity within freshwater as compared to marine habitats, with extended longevity more common in the former than the latter. Nevertheless, the mean age of the lake populations studied here was similar to that of the marine populations (mean = 2.1). Similar trends were observed in size data: while most fish from the lake and marine habitats were between 35 and 45 mm, several lake individuals were more than 50 mm whereas this size class was not represented among the marine populations. Dryer [42] also found nine-spined sticklebacks ranging from 40 to 80 mm in Lake Superior, again reflecting the wide variation and large size that can be reached by freshwater nine-spined sticklebacks. Although the paucity of reports from marine nine-spined sticklebacks makes it difficult to determine the maximal range, our data suggest that it is not likely to reach lengths observed in freshwater habitats.

Age-at-maturation did not follow the same trend as longevity: first breeding most commonly appeared to take place at an age of two years – both in marine and lake populations, whereas no mature second year fish were recorded in most of the pond populations. Hence, differences in age-at-maturation were observed within freshwater habitats, with the pond fish generally maturing later than the lake fish. However, there are two notable exceptions (Krugloje; KRU and Mashinoje; MAS) where mature two-year-olds were found in ponds – both of which are situated close to the coast-line. Interestingly, these ponds are also exceptional in respect to their ecology and demography: in contrast to other pond populations, they harbor interspecific competitors ([30], Table S1), exhibit higher levels of genetic variability [43] and display much lower size-at-age than the other pond populations (this study; [30]). In these respects, these two populations appear to be ecologically and demographically more similar to lake and marine populations, as compared to isolated pond populations subject to reduced predation pressure by piscine predators. Therefore, differences in age-at-maturation appear to be related to reduced predation risk and perhaps overall habitat complexity, wherein the absence of predators allows individuals to partition their resources mainly towards growth in early years and hence delay maturation. Indeed, this has been confirmed through a series of common-garden experiments that the period of fast, early growth in pond fish lasts longer than that in marine fish, allowing them to reach larger size ([44], [45]) and mature later [44], [46]. Differences in age at first breeding were also reported by Coad & Power [32], who found reproductive one year olds within a Canadian river, yet noted the absence of similarly aged reproductive individuals in a nearby lake. Although the ecological characteristics of these systems were not quantified, it is possible that significant differences in predation and ecological complexity could also be responsible for differences in age-at-maturation in other regions of nine-spined stickleback population distribution. The same has been noted in three-spined sticklebacks: several studies of lake-stream pairs have indicated that while stream sticklebacks mature early at a small size, lake conspecifics appear to mature later at larger sizes and live longer [47], [48].

Comparison of the results of the present study to those obtained from a similar study of Fennoscandian three-spined sticklebacks (*Gasterosteus aculeatus*; [37]) reveal several interesting differences. First, while six to seven year old nine-spined sticklebacks were encountered in several different locations in this study, the oldest reported three-spined sticklebacks observed among samples covering the roughly the same geographic area were only five years old [37]. Interestingly, these were from marine habitats, highlighting a second difference between these two species in Fennoscandia: while the marine nine-spined sticklebacks were younger and smaller than their freshwater conspecifics, the opposite was to true in the case of three-spined sticklebacks [37]. However, eight-year-old three-spined sticklebacks have been reported from one freshwater location in British Colombia [41], [49], indicating that a high amount of variation in age structure must exist among three-spined stickleback – albeit on a larger geographic scale (see also: [47], [48]). An additional noteworthy difference is that DeFaveri & Merilä [37] found one nanistic population of three-spined sticklebacks exhibiting small size and prolonged longevity, while prolonged longevity in nine-spined sticklebacks was distinctly associated with large size. Hence, the overall generalization that can be drawn from comparing life histories of the two stickleback species in Fennoscandia is that each of the species show a great deal of interpopulational variation in age at maturation, longevity and size-at-age, but patterning of this variability across different habitat types differs between the species. Furthermore, the range of variation both in longevity and age-at-size is much more pronounced in the nine-spined as compared to the three-spined stickleback in this area.

Finally, many sources of error are associated with aging of fish via skeltochronological methods, and validation of age estimates is important [50]. Two detailed otholith studies [26], [31] of nine-spined sticklebacks have made fairly good job in validating the annuli formation as a reliable aging method in this species. In our study, we have assumed that that annuli formation occurred in a comparable fashion in different populations. This assumption could be violated if annuli formation is for instance temperature dependent [51], [52] as there is likely to be great deal of heterogeneity in temperatures experienced by different populations. Yet, all of the populations included to this study – albeit coming from different habitat types and latitudes – are subject clear-cut seasonal temperature (and growth) cycles which should result in similar annuli formation. Nevertheless, verification of the annuli formation by using capture-recapture methods [49], [50], or by correlating skeletochronologically determined ages with other markers of aging (e.g. teleomere length; [53]) would provide the ultimate proof that variation in annuli numbers across different populations reflect age differences. Likewise, larger sample sizes, repeated sampling of the study localities (and microhabitats within localities) in different times of the year should provide more refined picture about distribution of longevity in different habitat types.

As to the practical implications of our results, it may be instructive to note that knowledge of age structure within populations – and the variation among them – can be relevant in studies aiming to estimate genetically effective population sizes (N_e_) from allele frequencies in marker loci. Namely, estimation methods require either knowledge (e.g. [54]) or assumptions (e.g. [55]) to be made about the extent of generation overlap and age structure. Our results revealed variation in generation length among nine-spined stickleback populations, and although such differences are critical to accurately estimate N_e_, they could nonetheless be easily overlooked if generation length is assumed to be the same across all populations. Furthermore, as a rule of thumb, it is recommended that when estimating N_e_ from temporal shifts in allele frequencies, the temporal samples should be spaced at least three to five generations apart [55]. Hence, the results presented in this paper should be helpful in designing sampling schemes for studies aiming to estimate N_e_ in nine-spined sticklebacks. For instance, given the observed interpopulation variation in average age of breeding individuals (i.e. generation length), the optimal sampling strategy is likely to be different for different populations.

In conclusion, the results suggest that nine-spined sticklebacks in isolated pond populations exhibit delayed maturation and increased longevity as compared to their conspecifics living in lake and marine habitats. However, extended longevity beyond ages earlier reported from more southern populations was observed also in part of the studied lake and marine populations. The larger size-at-age in pond as compared to lake and marine populations aligns with the evidence from laboratory experiments [30], [56] suggesting that delayed maturation and extended growth period of the pond fish allow them to reach larger sizes. To which degree the extended longevity in pond populations is influenced by intrinsic factors (i.e. delayed senescence) remains a challenge to be investigated in studies to come, but reduced extrinsic mortality rate due to lack of piscine predation in ponds is a likely explanation to the increased longevity of pond fish.

## Supporting Information

Table S1 **Information on physical and biological characteristic of the study sites.**
(DOCX)
