# Supplementary material for: Geographic Variation in Age Structure and Longevity in the Nine-Spined Stickleback (Pungitius pungitius)
Source: PLoS One. 2014 Jul 15;9(7):e102660. doi: 10.1371/journal.pone.0102660 (PMC4099423; doi:10.1371/journal.pone.0102660)
Supplement: Table S1 — Information on physical and biological characteristic of the study sites. (DOCX) [file pone.0102660.s001.docx]

Table S1. Information on physical and biological characteristic of the study sites.

Location Code Habitat Surface area (ha) Max. depth (m) Predatory fish Competitors

Fiskebäckskil KRI Marine NA NA PF,EL,ST,SS,LS GA, +

Helsinki HEL Marine NA NA PF,EL,ST,SS,LS GA, +

Oulu OUL Marine NA NA PF,EL,ST,SS,LS GA, +

Bölesviken BÖL Marine NA NA PF,EL,ST,SS,LS GA, +

Levin Navolok LEV Marine NA NA PF,EL,ST,SS,LS GA, +

Pulmankijärvi PUL Lake 1620 35 PF,EL,ST,SS, TT GA, +.

Tuolpujärvi TUO Lake 185 < 15 TT, ? +

Iso-Porontima POR Lake 115 42 PF, EL, ST, TT +

Stavlussukjavri STA Lake 30 > 6 SA none

Pyöreälampi PYÖ Pond < 5 > 10 none none

Rytilampi RYT Pond < 5 5 none none

Kirkasvetinen lampi KIR Pond < 5 ? none none

Krugloje KRU Pond < 5 ? none GA

Mashinoje MAS Pond < 5 3 none GA

Abbortjärn ABB Pond < 5 ? none none

Bynastjärnen BYN Pond < 5 ? none none

PF = *Perca fluviatilis*, EL = *Esox lucius*, ST = *Salmo trutta*, SS = *Salmo salar*, LS = *Lucioperca sander*, TT = *Thymallus thymallus, SA = Salvelinus alpinus*

GA = *Gasterosteus aculeatus*, + = other species of fish feeding on zooplankton and benthic invertebrates. + = various other non-predatory fish species which may be competitiors to nine-spined sticklebacks, ? = information not available
